# Supplementary material for: Causal Relationship between Aging and Anorexia Nervosa: A White-Matter-Microstructure-Mediated Mendelian Randomization Analysis
Source: Biomedicines. 2024 Aug 16;12(8):1874. doi: 10.3390/biomedicines12081874 (PMC11351342; doi:10.3390/biomedicines12081874)
Supplement: Supplementary file 1 [file biomedicines-12-01874-s001.zip › Supplementary Figure/Figure S8/Leaveoneout plot of MR analysis for TL to ubm-b-1922.pdf]

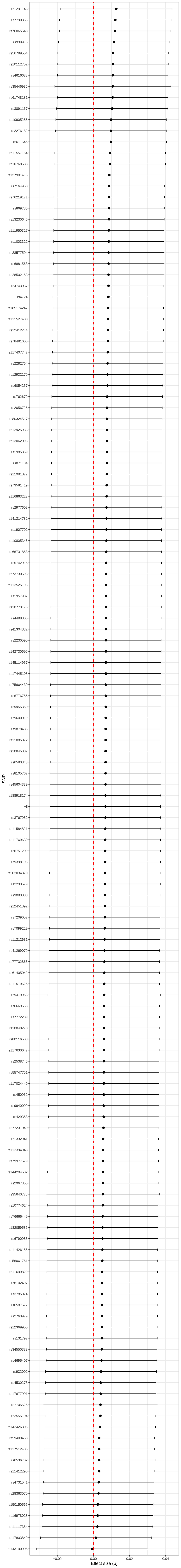

rs1291143
rs7790856
rs76065543
rs939916
rs56799554
rs10112752
rs4616688
rs35446936
rs61748181
rs3891167
rs10905255
rs2276182
rs611646
rs11557154
rs10768683
rs137901416
rs7164950
rs76219171
rs869785
rs13230646
rs111950327
rs1003322
rs28577594
rs6881568
rs28502153
rs4743037
rs4724
rs185174247
rs111527438
rs12412214
rs78491606
rs117407747
rs2282764
rs12932179
rs6054257
rs762679
rs2056726
rs80324517
rs12925933
rs13062095
rs1985369
rs871134
rs11991877
rs73581419
rs116863223
rs2977608
rs141214782
rs1907702
rs10805346
rs66731853
rs5742915
rs73730598
rs113525195
rs1957937
rs10773176
rs4498805
rs41304832
rs2230590
rs142730696
rs145114957
rs17445108
rs75664430
rs6776756
rs9955360
rs9600019
rs9878436
rs11085072
rs10845387
rs6590343
rs8105767
rs45604339
rs188918174
All
rs3767952
rs11584821
rs11769630
rs6751209
rs9398196
rs202034370
rs2293579
rs3093888
rs12451892
rs7209057
rs7099229
rs11212631
rs41269079
rs77732866
rs61405042
rs11579626
rs9419958
rs6669563
rs7772289
rs10840270
rs80116508
rs117630647
rs2538745
rs55747751
rs117034449
rs450962
rs9940099
rs429358
rs77231040
rs1332941
rs112394943
rs79977579
rs144204502
rs2967355
rs35640778
rs10774624
rs76666449
rs182059586
rs6790988
rs11426156
rs56061761
rs11699829
rs8102497
rs3785074
rs6587577
rs2763979
rs12369950
rs131797
rs34550383
rs4695407
rs932002
rs4530278
rs17677991
rs7705526
rs2555104
rs142426306
rs59409453
rs117512405
rs6536702
rs11412296
rs4731541
rs28363070
rs150150565
rs16978028
rs11117354
rs17803849
rs143190905
SNP
−0.02
0.00
0.02
0.04
Effect size (b)
